# Successful surgical correction of an incomplete atrioventricular septal defect in a 76-year-old female patient

**DOI:** 10.1093/jscr/rjae187

**Published:** 2024-03-27

**Authors:** Kentaro Shirakura, Nobuyuki Akasaka, Daichi Mizushima, Masahiko Narita, Ryo Okubo, Tomoki Nakatsu, Daita Kobayashi, Hiroyuki Kamiya

**Affiliations:** Department of Cardiovascular Surgery, Steel Memorial Muroran Hospital, Chiribetsucho 1-45, Muroran 050-0076, Japan; Department of Cardiovascular Surgery, Steel Memorial Muroran Hospital, Chiribetsucho 1-45, Muroran 050-0076, Japan; Department of Cardiovascular Surgery, Steel Memorial Muroran Hospital, Chiribetsucho 1-45, Muroran 050-0076, Japan; Department of Cardiovascular Surgery, Steel Memorial Muroran Hospital, Chiribetsucho 1-45, Muroran 050-0076, Japan; Department of Cardiac Surgery, Asahikawa Medical University, Midorigaoka Higashi 2-1-1-1, Asahikawa 078-8510, Japan; Department of Cardiovascular Surgery, Steel Memorial Muroran Hospital, Chiribetsucho 1-45, Muroran 050-0076, Japan; Department of Cardiovascular Surgery, Steel Memorial Muroran Hospital, Chiribetsucho 1-45, Muroran 050-0076, Japan; Department of Cardiac Surgery, Asahikawa Medical University, Midorigaoka Higashi 2-1-1-1, Asahikawa 078-8510, Japan

**Keywords:** left atrioventricular valve, mitral valve repair, patch closure

## Abstract

We report the case of a 76-year-old woman with an incomplete atrioventricular septal defect and severe congestive heart failure who underwent surgical repair. Surgical intervention involved mitral valve repair and patch closure of the ostium primum defect, resulting in a favorable postoperative course. Successful outcomes support surgery as a reasonable treatment option owing to its significant improvement in postoperative quality of life, even in elderly patients with left atrioventricular valve degeneration.

## Introduction

Atrioventricular septal defects (AVSDs) are categorized into two main types: complete and incomplete defects. An incomplete AVSD encompasses a primary septal defect within the atrial septum and a cleft in the mitral valve, also referred to as an ostium primum defect. In adults, a substantial number of surgical cases related to incomplete AVSDs have consistently favorable outcomes, even during long-term follow-up. Surgical interventions for patients aged over 70 years are rare [1, 2]. Here, we describe the surgical repair of an incomplete AVSD in a 76-year-old woman with severe congestive heart failure.

## Case report

**Figure 1:**
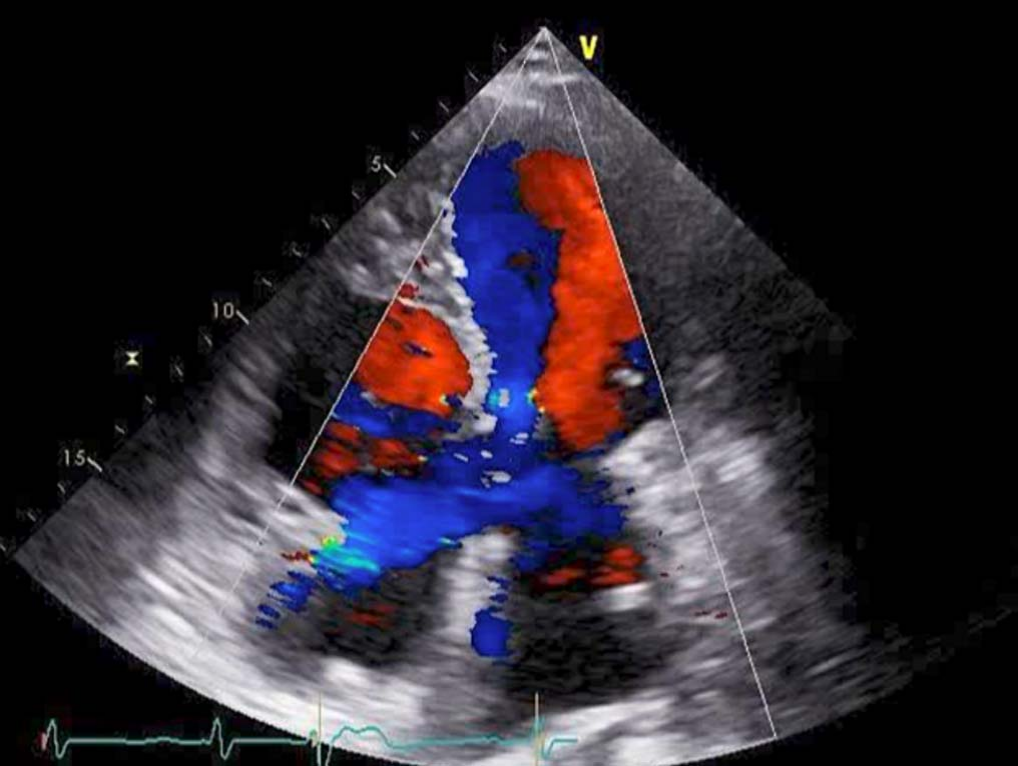
Preoperative cardio echography showing fraction EF 65%, MR severe, mean PAP 32 mmHg, left-to-right shunt flow, LVDd 48 mm, LVDs 32 mm, and a cleft mitral valve.

**Figure 2:**
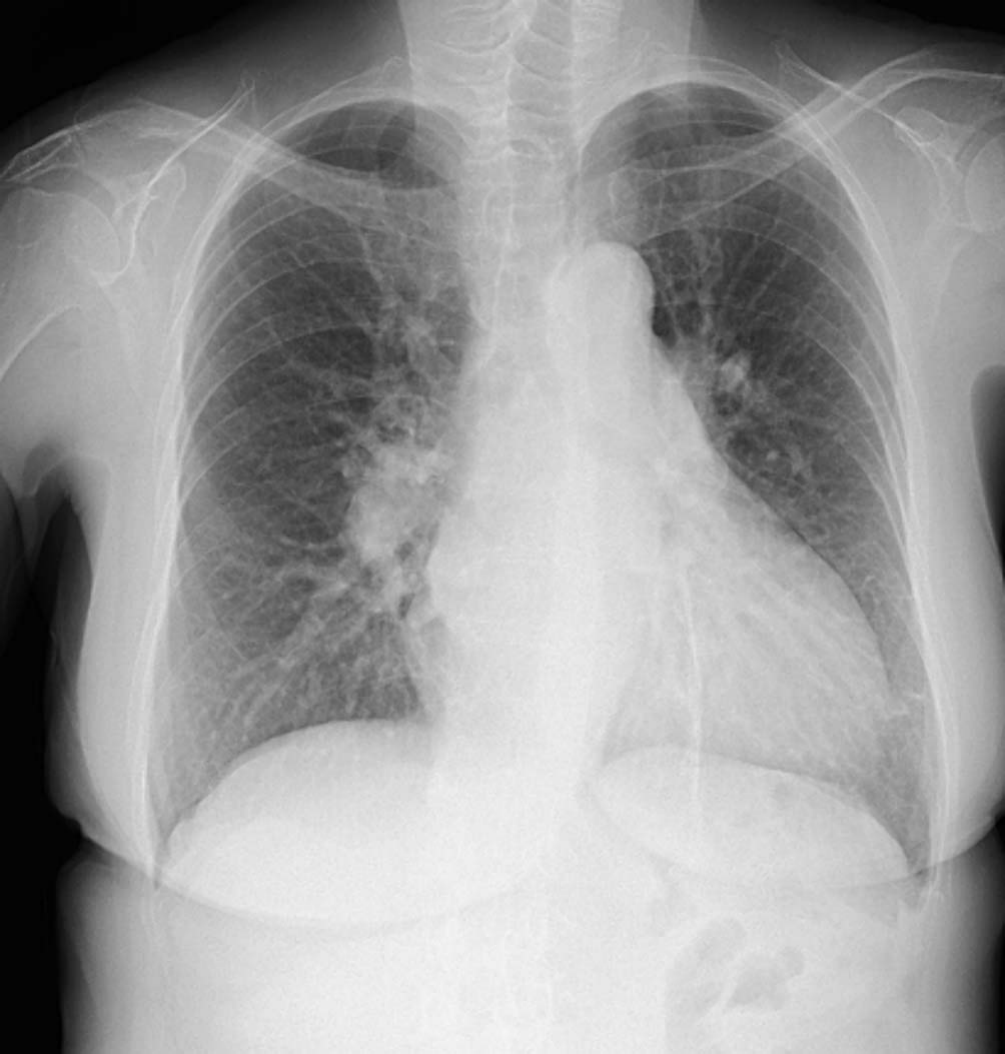
Chest X-ray showing a CTR of 58% and CPA sharp.

The patient was followed up as an outpatient since 2010 for incomplete AVSD and congestive heart failure by the Cardiovascular Surgery Department at our hospital. She did not wish to undergo surgery; therefore, we continued to follow her as an outpatient. Mitral regurgitation (MR) III-IV and mitral valve deviation was detected by echocardiography at approximately 2022, with worsening heart failure symptoms (New York Heart Association (NYHA) class III status). Echocardiography revealed an ejection fraction (EF) of 65%; severe MR; mean pulmonary artery pressure (PAP) of 32 mmHg; left-to-right shunt flow; left ventricular dysfunction (LVDd) of 48 mm; LVDs, 32 mm; and cleft mitral valve (Fig. 1). Chest radiography revealed a cardiothoracic ratio (CTR) of 58% and a costophrenic angle (CPA) sharp (Fig. 2). Cardiac catheterization revealed a Qp/Qs ratio of 2.6. Therefore, we performed a mitral valve repair and patch closure of the primum ostium defect.

**Figure 3:**
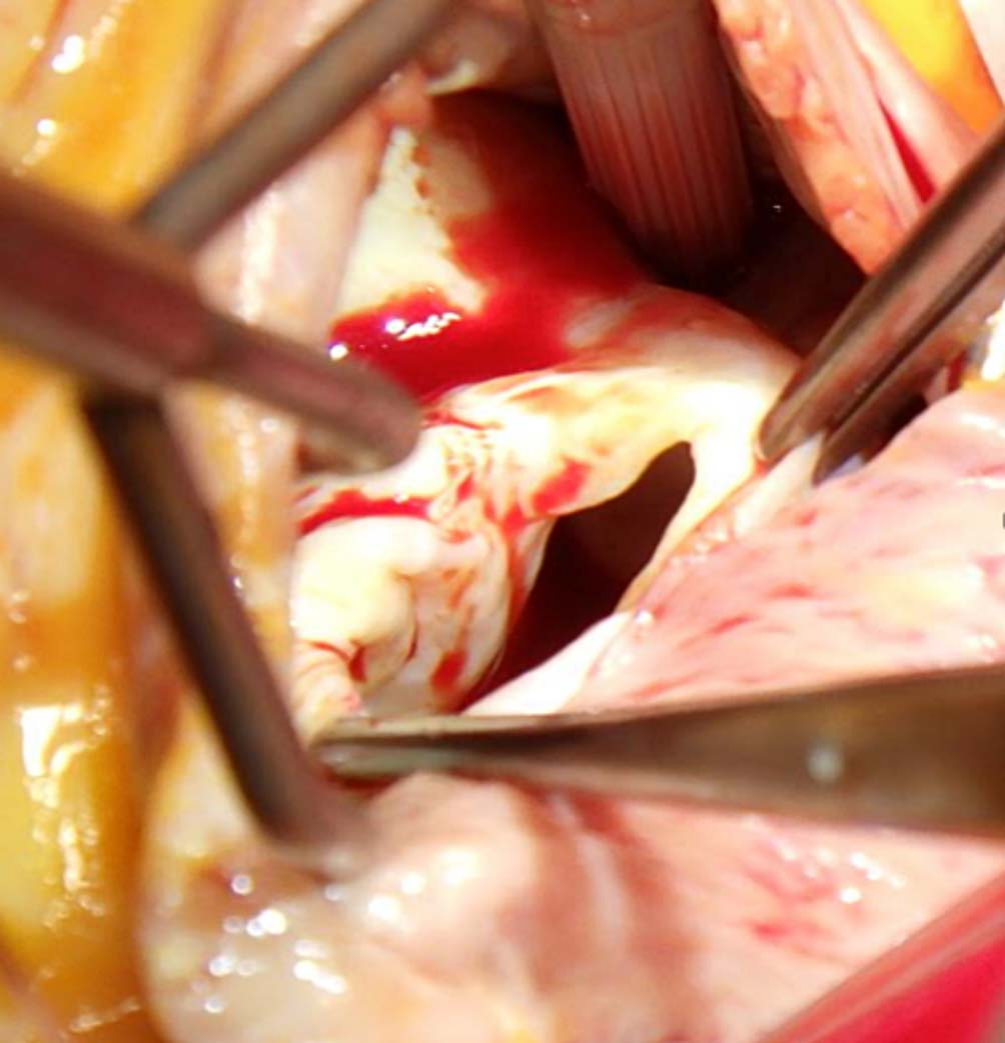
Ostium primum defect observed from the right atrium side.

The surgery was performed through a median sternotomy with arterial cannulation of the ascending aorta and venous cannulation of the vena cava. After cardioplegic arrest, a right atrial incision was made and the ostium primum defect was confirmed (Fig. 3). The atrial septal defect was extended, and rupture of the two marginal chordae of mitral valve P3 was observed. Three CV4 (Gore-tex, Gore Medical, NJ) 22 mm loops were used as neochordae from the posterior papillary muscle to the P3 segment. An annuloplasty band (Tailor Band, 27 mm; Abbott, IL, USA) was used. The ostium primum was sutured with an autologous pericardial patch on the tricuspid valve side and the coronary sinus was retained on the left atrial side.

**Figure 4:**
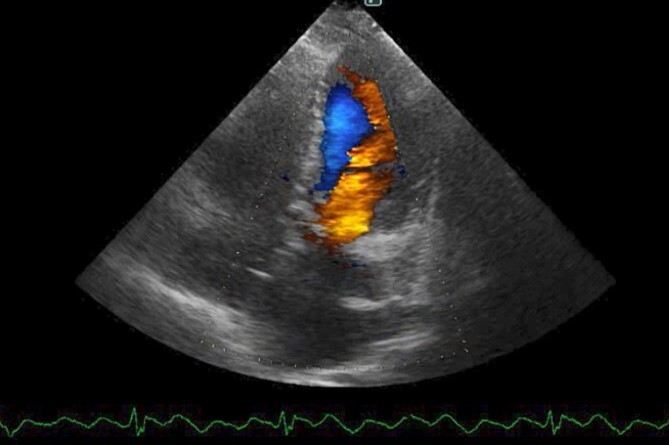
Postoperative cardio echography showing an EF of 64%, trivial MR, mean PAP of 18 mmHg, and no shunt flow.

The patient had an uneventful postoperative course during her hospital stay. Echocardiography on postoperative Day 8 showed an EF of 64%, trivial MR, a mean PAP of 18 mmHg, and no shunt flow (Fig. 4). Therefore, the patient was discharged on the 19th day after surgery. The patient is doing very well 7 months after surgery.

## Discussion

There are many surgical cases of incomplete AVSD in adults, and good results are reported. The prognosis is not significantly different from that of secundum atrial septal defect (ASD) [1, 3]. The conditions for long-term survival include: (i) simple cardiac malformation with only an ostium primum atrial septal defect or a cleft in the atrioventricular valve with minimal valve dysfunction; (ii) maintenance of sinus rhythm and no permanent arrhythmia until a relatively old age; (iii) mild cardiac dysfunction and late onset of heart failure; (iv) no other concomitant cardiac malformations or simple malformations; and (v) no severe pulmonary hypertension [1, 4, 5]. In addition to these conditions, the patient continued medical treatment (including blood pressure control) before the onset of heart failure, which was considered to be one of the factors contributing to long-term survival.

Surgery cases in patients aged 70 years are rarely reported. To the best of our knowledge, only a few cases of surgery in patients aged >70 years of age have been reported [1, 2]. Postoperative complications of AVSDs depend on the presence of atrioventricular regurgitation, a factor known to influence prognosis [6, 7]. In our case, the postoperative heart failure improved to NYHA class I, and pulmonary hypertension markedly improved. Postoperative echocardiography revealed no atrioventricular regurgitation. These findings suggest that elderly patients with mild degeneration of the left atrioventricular valve degeneration may benefit from valvuloplasty. Late postoperative complications of AVSDs depend on residual atrioventricular regurgitation (especially in elderly patients), although new cases of ventricular dysfunction, severe atrioventricular block, or atherosclerotic lesions may develop over time. Therefore, regular follow-up is necessary in the future.

## Conclusion

A 76-year-old patient with an incomplete AVSD who presented with symptoms of heart failure underwent surgery. The patient’s postoperative course was uneventful and she was discharged on Day 19. The patient is doing very well 7 months after surgery. Valvuloplasty is considered a reasonable treatment option owing to its excellent results and significant improvement in postoperative quality of life, even in elderly patients with left atrioventricular valve degeneration.
